# Impact of lipid emulsions in parenteral nutrition on platelets: a literature review

**DOI:** 10.1017/jns.2024.11

**Published:** 2024-03-25

**Authors:** Betul Kisioglu, Funda Tamer

**Affiliations:** 1 Hacettepe University, Faculty of Health Sciences, Department of Nutrition and Dietetics, Ankara, Turkey; 2 Duzce University, Faculty of Health Sciences, Department of Nutrition and Dietetics, Duzce, Turkey

**Keywords:** Lipid emulsions, lipids, parenteral nutrition, platelet function tests, platelets

## Abstract

Lipid emulsions are essential components of parenteral nutrition solutions that provide energy and essential fatty acids. The complexity of the formulations of lipid emulsions may lead to adverse outcomes such as platelet reactivity and changes in platelet aggregation and related coagulation. Platelets are responsible for haemostasis; they activate and demonstrate morphological changes upon extracellular factors to maintain blood fluidity and vascular integrity. Although parenteral nutrition lipid emulsions are generally found safe with regard to modulation of platelet activity, studies are still accumulating. Thus, this review aims to investigate platelet-related changes by parenteral nutrition lipid emulsions in human studies. Studies have pointed out patients at risk of bleeding and increased platelet aggregation responses due to the administration of lipid emulsions. Lipid emulsions may further benefit patients at high risk of thrombosis due to anti-thrombotic effects and should be cautiously used in patients with thrombocytopenia. The reported platelet-related changes might be associated with the fatty acid change in the plasma membranes of platelets following changes in platelet synthesis and plasma levels of eicosanoids. In conclusion, studies investigating platelets and parenteral nutrition should be supported to minimize the adverse effects and to benefit from the potential protective effects of parenteral nutrition lipid emulsions.

## Graphical abstract

**Figure:**
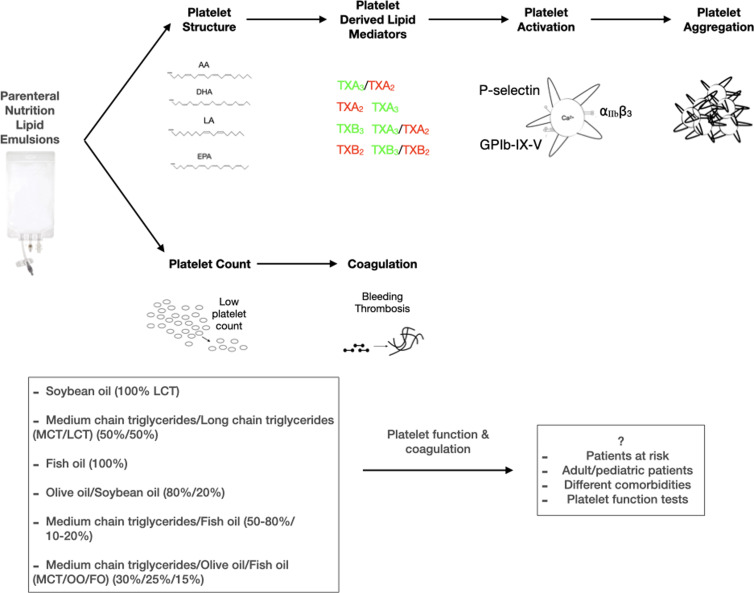

## Introduction

Lipid emulsions are vital components of parenteral nutrition (PN) solutions^(1–3)^ and provide about 25-40% PN non-protein energy^(4,5)^, prevent essential fatty acid deficiency^(4,6,7)^ and may have anti-inflammatory, or immune-modulating properties^(8,9)^. The triglyceride (TG) component of lipid emulsions may be from different sources, including soybean oil (SO), olive oil (OO), safflower oil, coconut oil, and fish oil (FO)^(4,10)^. First (SO), second (SO + medium-chain triglycerides)/third (OO), and fourth generation (FO) lipid emulsions can be identified as pro-inflammatory, inflammatory neutral, and anti-inflammatory, respectively^(4,10)^.

Commercially available lipid emulsions contain primarily long-chain triglycerides (LCT) along with mixes, including medium-chain triglycerides (MCT), which are referred to as MCT/LCT^(5)^. Soybean oil-based emulsions contain 44-62% of linoleic acid (LA) (Table 1)^(2,11)^ and may have pro-inflammatory effects via eicosanoids synthesized from n-6 PUFA arachidonic acid (AA)^(11)^. Olive oil is reported to be more advantageous than SO because of its higher MUFA and lower n-6 PUFA content (Table 1)^(11)^. Similarly, FO lipid emulsions are used for their proposed anti-inflammatory and immunomodulatory effects^(2,3,6,7,12)^. These lipid emulsions may be used in combination with other lipid sources (SO, MCT, OO, and FO-containing lipid emulsions) to lower the amount of SO. Furthermore, they contain higher n-3 PUFA, α-tocopherol, and low amounts of plant phytosterols than SO-based lipid emulsions (Table 1)^(11)^.

**Table 1.** Comparison of lipid emulsions in lipid source and fatty acid composition

	Lipid Emulsion					
	SO (100% LCT)	MCT/LCT (50%/50%)	FO (100%)	OO/SO (80%/20%)	MCT/FO (50%/10%)	MCT/OO/FO (30%/25%/15%)
Lipid source	Soybean oil	Soybean oil, MCT^b^	Fish oil	Soybean oil, olive oil	Soybean oil, MCT^b^, fish oil	Soybean oil, MCT^b^, olive oil, fish oil
Fatty Acid Percentage (%)^a^						
Caproic acid (6:0)	–	0.5	–	–	–	–
Caprylic acid (8:0)	–	26-28.5	–	–	30	13-24
Capric acid (10:0)	–	10-20	–	–	19.5	5-15
Lauric acid (12:0)	–	1	–	–	–	–
Myristic acid (14:0)	–	–	2-7	–	0.5	1
Palmitic acid (16:0)	7-14	7.5	4-12	7.6-19.3	6	7-12
Palmitoleic acid (16:1)	–	–	4-10	1.5	0.5	1.5
Stearic acid (18:0)	1.4-5.5	–	–	0.7-5.0	–	1.5-4
Oleic acid (18:1)	19-30	11-14	4-15	44.3-79.5	8	23-35
Linoleic acid (18:2)	44-62	48.0-58.0	1.5-4.5	13.8-22.0	38.4-46.4	14-25
α-Linolenic acid (18:3)	4-11	5.0-11.0	1.1-1.8	0.5-4.2	4.0-8.8	1.5-3.5
Arachidonic acid (20:4)	–	–	0.2-2.0	–	–	–
EPA (20:5)	–	–	13-26	–	3.5	1-3.5
DPA (22:5)	–	–	2	–	3	–
DHA (22:6)	–	–	14-27	–	2.5	1-3.5
SFA	15	58	21	14	49	37
MUFA	24	11	23	64	14	33
PUFA						
n-3	8	4	48	3	10	7
n-6	53	27	5	19	27	23
n-6:n-3 ratio^c^	7:1	7:1	1:8	9:1	2.7:1	2.5:1

SO, soybean oil; MCT, medium-chain triglycerides; LCT, long-chain triglycerides; FO, fish oil; OO, olive oil; EPA, eicosapentaenoic acid; DPA, docosapentaenoic acid; DHA, docosahexaenoic acid; MUFA, monounsaturated fatty acids; PUFA, polyunsaturated fatty acids.

–Not detected or not reported (in either situation, there is little or none present).

a Data provided is from the manufacturer data sheets or^(7, 12)^.

b MCT lipid sources may be coconut, palm kernel or other tropical nut oils.

c Data taken from^(96)^.

**Fig. 1.**
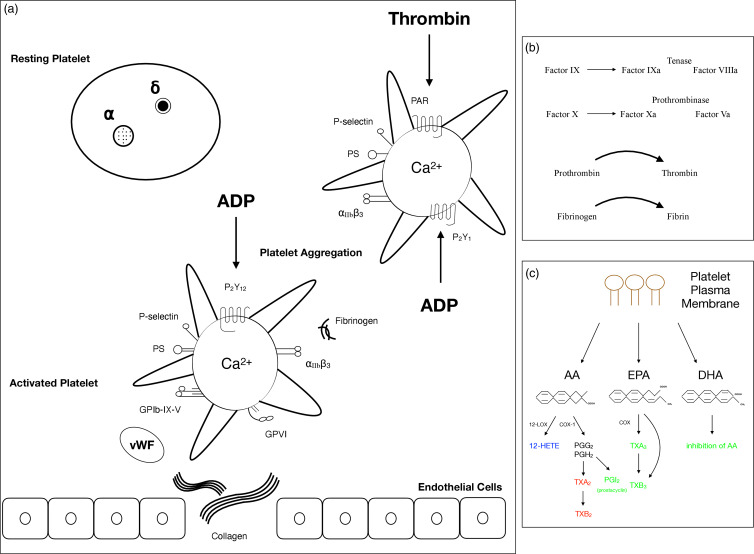
a. Signalling mechanisms during platelet activation. A resting platelet owns a discoid shape with α and δ granules, and an activated platelet becomes round and forms pseudopods. Strongly activated platelets have high cytosolic Ca^2+^. Collagen is exposed from the damaged endothelium and binds to GPVI and GPIb-IX-V with another ligand vWF. Fibrinogen activates the platelet through the integrin α_IIb_β_3_ (GPIIb/IIIa). Integrin activation leads to platelet aggregation. Agonists ADP and thrombin take roles through receptors P_2_Y_1_, P_2_Y_12_, and PAR. Activated platelets express PS on their surface. An adhesive molecule, P-selectin, is expressed on an activated platelet. b. Scheme of the coagulation cascade. Steps into the coagulation cascade: fibrinogen produces fibrin through thrombin. Fibrin is responsible for forming a tight thrombus. c. Scheme of the produced lipid mediators via the platelet plasma membrane. Lipid mediators form through the release of platelet membrane phospholipid fatty acids, which may affect platelet activation/aggregation. 12-HETE may increase or decrease platelet activation. TXA_2_ and TXB_2_ are pro-coagulants and increase platelet activation. TXA_3_ reduced platelet activation, whereas endothelial cell-derived PGI_2_ inhibits platelet activation. DHA is known to inhibit AA formation in human platelets. AA, arachidonic acid; ADP, adenosine diphosphate; COX, cyclooxygenase; DHA, docosahexaenoic acid; EPA, eicosapentaenoic acid; LOX: lipoxygenase; PG, prostaglandin; PS, phosphatidylserine; TX, thromboxane; vWF, von Willebrand factor; 12-HETE, 12-hydroxyeicosatetraenoic acid.

Parenteral nutrition may influence haemorheological parameters and platelet function^(13)^, and it is now well established that these complications may result from the lipid source of PN solutions. Lipid emulsions will likely affect platelet function due to the many biological functions (inflammatory and immune responses, coagulation, cell signalling)^(2,14–16)^. Platelets, the most abundant cells in the blood, are the vital cells responsible for haemostasis^(17,18)^. The functional changes of platelets (adhesion, activation, spreading, secretion, aggregation, pro-coagulant activity, microparticle formation, clot retraction) followed by tissue damage or other pathophysiological conditions (atherosclerotic lesion, inflammation) exist to maintain haemostasis^(19,20)^. Impaired platelet function and abnormal platelet number may result in bleeding or thrombus formation^(19,20)^. A group of agonists activates platelets in physiological haemostasis and pathological bleeding or thrombosis, and a cascade of reactions occurs. It is important to note that increased responsiveness of platelets to agonists in conditions like endothelial damage or pro-inflammatory state over-activates platelets in favour of a thrombotic state^(21)^ (Fig. 1).

Platelet function tests to monitor the risk of bleeding or predict thrombosis are essential in the hospital^(19,20)^. Traditional tests such as bleeding time and aggregometry are widely used^(19,20)^. Partial thromboplastin time (PTT) and prothrombin time (PT) are basic clotting tests used to exclude coagulation defects^(19,20,22)^. As the gold standard of platelet function^(20)^ aggregometry tests that give responses to a panel of agonists^(19,20)^ are still used; however, these tests are proposed not to define responses to weak agonists on a clinical basis^(19,20)^. Platelet function tests such as flow cytometry can explore the assessment of signalling processes and activation properties^(19,20,23)^. Flow cytometry and cell morphology under the microscope can measure platelet activation, such as integrin activation, secretion of granule contents, and phosphatidylserine (PS) exposure^(23)^. Platelet glycoproteins, activation markers, and platelet function are investigated^(19,20)^. Moreover, studies also investigate the phospholipid composition of platelet plasma membranes that is tightly regulated for haemostasis^(24)^ and can also signify platelet activation; for example, PS is exposed highly on the surface before coagulation^(24)^ (Fig. 1).

Thrombocytopenia, platelet dysfunction, hypercoagulation, and bleeding were reported in patients when lipid emulsions were introduced in PN solutions. Thromboembolic events, including thrombosis, thromboembolism^(25–29)^, major bleeding^(30)^, vena cava syndrome, thrombocytopenia, and heparin complications^(31)^, are seen in many patients due to PN. During hospital stays, patients are prone to thromboembolic events due to reduced physical activity^(32)^. PN might affect haemostasis and could potentially increase the risk of bleeding or postoperative thrombosis. Also, PN is used in patients (critically ill, patients on chemotherapy, bone marrow transplantation) who are likely to develop thrombocytopenia^(30)^. Intravenous (IV) fat emulsion infusions have been associated with bleeding complications, thrombocytopenia, effects on cholesterol, triglyceride, glucose, insulin and blood pressure^(33)^.

On the other hand, lipid emulsion infusion may be used for the benefit of patients to prevent preoperative infarction, postoperative occlusion of coronary arteries^(34)^, and severe hypercoagulability cases^(35)^. Potentially, some lipid emulsions may have anti-atherogenic properties^(36,37)^, and these effects should be defined to be used in patients with accelerated atherosclerosis as the inhibition of platelet aggregation could lead to arrest or regression of atherosclerotic process^(36)^.

The influence of PN lipid emulsions on platelet function and coagulation is a controversial issue^(32)^. Although ESPEN defines lipid emulsions as generally safe concerning platelet activity^(38,39)^, there needs to be more knowledge on the effects of PN solution contents on normal platelet functions, as well as in bleeding and thrombotic disease processes. Platelet activation plays a central role in the pathogenesis of thromboembolic incidents^(32)^, and platelet dysfunction is a significant cause of excessive bleeding^(34)^. Therefore, the PN treatment should not lead to platelet aggregation, which might lead to atherosclerotic lesions and acute thrombosis^(32)^, or platelet dysfunction and bleeding^(34)^. In this review, we gathered and summarized studies investigating parenteral nutrition lipid emulsions and their effects on platelets in order to provide clinically-relevant updates on parenteral nutrition and haemostasis.

## Methods

This review gathered human studies investigating parenteral nutrition lipid emulsions and their effects on platelets. PubMed, Science Direct, Google Scholar, and Scopus databases were used with the keywords ‘lipid emulsion/parenteral nutrition and platelets’. Due to limited research, all studies (1971–2022) conducted on human subjects (n = 51) were included, and studies on experimental animals were excluded.

**Fig. 2.**
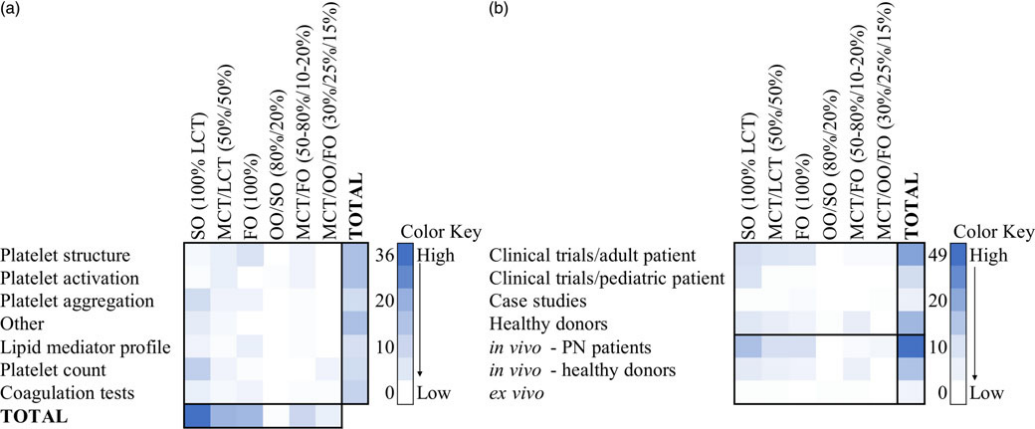
Heat map of studies investigating parenteral nutrition lipid emulsion on platelets distributed to a. platelet function tests b. type of research. FO, fish oil; LCT, long-chain triglycerides; MCT, medium-chain triglycerides; OO, olive oil; PN, parenteral nutrition; SO, soybean oil.

The impact of parenteral nutrition lipid emulsions on platelets was divided into two headings that are (1) platelet function and (2) platelet count and coagulation. Further, studies were investigated by subheadings (1) soybean oil-based, (2) soybean oil/medium-chain triglycerides-based, (3) fish oil-based, (4) olive oil/soybean oil-based, (5) medium-chain triglycerides/fish oil-based, (6) soybean oil/medium-chain triglycerides/olive oil/fish oil-based. Due to insufficient literature, some subheadings were evaluated together (Fig. 2).

## Impact of PN lipid emulsions on platelet function, platelet count, and coagulation

### Soybean oil-based lipid emulsion

#### Impact on platelet function

The most studied lipid emulsion in the literature in the context of platelets is soybean oil-based lipid emulsion (100% LCT) (Fig. 2). To start with, no change was reported in platelet aggregation in infants^(40)^ and adult patients^(30,41,42)^ with SO (100% LCT) lipid emulsion administration. Interestingly, it is known that SO lipid emulsions contain the precursor (linoleic acid) of arachidonic acid, which, even in low concentrations, might activate and induce platelets to undergo a release reaction and aggregate^(30)^. Despite this, there was no clinical evidence of bleeding or thrombotic tendency in these patients receiving SO lipid emulsion^(41)^. Further, the lipid emulsions may also lead to increased plasma levels of non-esterified fatty acids (NEFA), associated with platelet aggregation^(40,41)^. In contrast, decreased platelet aggregation was reported in familial hypercholesterolaemia patients receiving SO (100% LCT) infusion^(36)^. Since enhanced platelet aggregation is seen in patients with accelerated atherosclerosis thus, inhibition of platelet aggregation points to potential anti-atherosclerotic effects of SO (100% LCT) and may be due to specific diseases and patients^(36,37)^ (Table 2).

**Table 2.** Summary of the studies measuring the effect of soybean oil-based lipid emulsions on platelet function

Platelet Test	Population	Duration	Main Platelet-Related Outcome	Reference
Platelet Structure	Critically ill adult patients (n = 12)	7 days	↑ LA, ↓ caprylic acid, DHA	^(45)^
	Patients with malnutrition (n = 20)	8-10 days	↑ LA, ↓ Oleic acid percentage Lauric and myristic acids not detected in the platelet phospholipids	^(46)^
Platelet Aggregation	Infants on PN (n = 10) Healthy donors (n = 10)	16 h infusion (1 g/kg)	= platelet aggregation, before and after SO (100% LCT) = platelet aggregation by SO (100% LCT)*, ex vivo* experiments (healthy donors)	^(40)^
	IV nutrition patients (n = 6)	app. 9-23 days	= platelet aggregation Lower concentrations of sodium arachidonate required to induce platelets to aggregate and to undergo a release reaction	^(41)^
	Patients with severe Familial Hypercholesterolaemia (n = 5) Control subjects (n = 4)	6 h infusion after 14 h fasting	↓ (50%) *in vitro* platelet aggregation by collagen during the infusion in comparison to pre-infusion values	^(36)^
	Patients (inflammatory bowel disease) (n = 20) Healthy controls (n = 23)	8-10 days	= platelet aggregation of patient’s pre- and post-PN results = platelet aggregation post-SO (100% LCT) and post-MCT/LCT (50%/50%) = platelet aggregation pre-PN and post-PN.	^(30)^
	Critically ill adult patients (n = 23)	7 days	= platelet aggregation SO (100% LCT) and MCT/LCT (50%/50%)	^(42)^
	Healthy male volunteers (n = 8)	> 3 h constant infusion (250 mL)	= *ex vivo* platelet aggregation = platelet morphology Internalization of small lipid particles in circulating platelets.	^(44)^
	Healthy male donors (n = 10)	infusion over 4 h after overnight fasting	↓ post-infusion *in vitro* platelet aggregation in response to ADP, the peak rate by 31%, and the 1 min. rate by 21%. The rates increased again, reaching the pre-infusion levels 20 hours later.	^(43)^
	Healthy donors (n = 20)	infusion 5 h after overnight fasting	↑ (slightly) post-infusion platelet aggregation in the presence of TRAP and collagen; however, NS ≤ TRAP and collagen-induced platelet aggregation pre-infusion but to a lesser degree post-infusion (mitochondrial inhibitors)	^(33)^
	Healthy male donors (n = 5)	6 h infusion after overnight fasting	≤ *in vitro* platelet aggregation induced by collagen or ADP ≤ platelet-free cholesterol content during the infusion period 18 h after the end of the infusion, platelet aggregation and cholesterol content returned to pre-infusion levels.	^(37)^
Platelet Activation	Healthy donors (n = 10) *ex vivo*	3 h citrated blood	< platelet adhesion returns to pre-infusion values after infusion > platelet adhesion	^(35)^
Platelet Activation Parameters	Critically ill adult patients (n = 23)	7 days	↑ PF4 (platelet-activating factor 4) and βTG (beta-thromboglobulin) continued to be elevated at 4-7 days of PN	^(42)^
	Healthy male volunteers (n = 8)	> 3 h constant infusion (250 mL)	↑ plasma βTG (beta-thromboglobulin), PF4 (platelet factor 4), serotonin (5-HT).	^(44)^
Lipid Mediator Profile	Septic shock patients (n = 10) and healthy controls (n = 8)	10 days	↓ PAF secretion TXA_3_/TXA_2_ ratio remained below 1% - SO (100% LCT) and control	^(49)^
	Patients with chronic plaque-type psoriasis skin disease (n = 83)	14 days	= 3-series TX = AA (plasma)	^(80)^
	Critically ill adult patients (n = 23)	7 days	↓ 6-keto-PGF_1α_ values after 4-7 days, NS	^(42)^

SO, soybean oil; LCT, long-chain triglycerides; LA, linoleic acid; DHA, docosahexaenoic acid; MCT, medium-chain triglycerides; ADP, adenosine diphosphate; TRAP, thrombin receptor activator peptide; NS, non-significant; PAF, platelet-activating factor; TX, thromboxane; FO, fish oil; AA, arachidonic acid; PG, prostaglandin.

↑, increase; ↓, decrease; =, no change; ≤, reduce.

Studies investigating healthy subjects have shown different results. Decreased platelet aggregation responses to agonists were also shown in healthy male donors^(37,43)^ due to SO (100% LCT) lipid infusion. These studies also indicated anti-atherogenic properties of SO (100% LCT) lipid emulsion due to reduced free cholesterol in mononuclear cells^(43)^ and platelets^(37)^, which might decrease platelet aggregation. The liposomes and triglyceride-phospholipid particles in SO (100% LCT) were held responsible for the change in platelet cholesterol content and *in vitro* platelet aggregation^(37)^ as they can lead to the depletion of cholesterol in cells and blood vessel endothelium^(43)^. Additionally, reduced platelet adhesion by SO (100% LCT) lipid emulsion infusion to healthy donors^(35)^ was shown; however platelet adhesion increased *ex vivo*. This study concludes that a humoral factor can reduce platelet adhesion. The slight decrease in platelet adhesion concluded that this lipid emulsion should not be infused in cases with severe hypercoagulability. In general, this lipid emulsion is safe to use without a risk of hypercoagulability^(35)^ (Table 2).

Other studies on healthy volunteers receiving SO (100% LCT) lipid infusion showed no change in platelet aggregation^(33,44)^. However, some subjects tended towards an increase in threshold values of platelet aggregation of some agonists, and there was a release of platelet-specific peptides such as βTG (beta-thromboglobulin), PF4 (platelet-activating factor 4), and 5-HT (5-hydroxytryptamine). The release of platelet products in the plasma and no sign of platelet activation under the electron microscope indicate different platelet subpopulations. Although normal platelet morphology was seen under the electron microscope, the lipid infusion showed internalized small lipid particles on the surface of platelets. These findings suggests that platelets are in close contact with lipid emulsion particles, which may induce changes in platelet surface properties, leading to electrochemical charge and rapid clearance of platelets from the circulation^(44)^. Additionally, mitochondrial inhibitors used in one study have led to limited ATP production eventually, leading to reduced platelet aggregation because platelets do not have enough energy to undergo shape change and granule release. However, these inhibitors were less effective post-infusion of SO (100% LCT), which may indicate promotion of aggregation at some point. Thus, SO (100% LCT) lipid emulsion might increase platelet aggregation when mitochondria function is inhibited and can affect the transcript profile of platelets with up-or down-regulation of genes associated with cell motility, adhesion, cell cycle progression, and metabolism. Continuous exposure would significantly solidify these changes and negatively impact haemostasis by increasing the risk for thrombosis^(33)^ (Table 2).

Accordingly, studies investigated the effects of PN lipid emulsions on platelet plasma membrane phospholipids. For instance, SO (100% LCT) lipid emulsion increased platelet LA and AA levels^(45,46)^ and conversely decreased oleic acid, caprylic acid, and docosahexaenoic acid (DHA) levels^(45)^. However, lauric acid and myristic acids were not detected in platelet phospholipids^(46)^. These fatty acid changes in platelet membranes may be due to lipid emulsions’ exogenous fatty acid source^(41)^. Additionally, the platelet fatty acid profile may change following differences in the plasma fatty acids. After SO (100% LCT) lipid emulsion administration, increased plasma of AA^(41)^, LA, palmitoleic, and palmitic acid levels, and decreased oleic acid levels were reported^(46)^ (Table 2).

The increased LA (precursor of AA), decreased DHA^(45)^, α-linolenic acid (ALA), along with a decreased ratio of n-3/n-6 PUFA content of the platelet membrane^(45)^, may also lead to platelet activation via eicosanoid synthesis^(47,48)^. Following the phospholipid fatty acid change in the platelet plasma membrane, the lipid mediator profile change is shown in a low TXA_3_/TXA_2_ ratio by SO (100% LCT)^(49)^, which may also lead to platelet activation and aggregation. Additionally, SO (100% LCT) increased PF4 and βTG in activated platelets^(42,44)^, indicating increased platelet activation (Fig. 2b).

#### Impact on platelet count and coagulation

Platelet count is a common blood test used in many studies investigating PN (Fig. 2a). Decreased platelet count, defined as thrombocytopenia, may increase risk of bleeding^(20)^. Thrombocytopenia was reported in patients (including children) receiving SO (100% LCT) along with morphologically abnormal large platelets^(50)^, decreased platelet survival^(51)^, and cholestatic liver disease^(52)^. Decreased platelet counts over time, and a higher risk of bleeding were reported to be associated with children with intestinal failure-associated liver disease (IFALD) that received SO (100% LCT)^(53)^.

In other studies, SO (100% LCT) administration did not cause thrombocytopenia^(33,35,54–56)^ in adult patients^(55)^, healthy donors^(35)^, and term and preterm newborns^(56)^. It is important to note that this may be due to phytosterols in lipid emulsions, which correlate with high plasma phytosterol levels. Also, reducing lipid infusion improved platelet count and liver function tests in some patients^(52)^. Further, high serum lipid levels may lead to fat overload syndrome with haematologic symptoms such as prolonged bleeding time and decreased platelet survival^(40)^. Interestingly, SO (100% LCT) administration may stimulate thrombocytopenia with more extended study duration periods^(56)^ (Table 3).

**Table 3.** Summary of the studies measuring the effect of different lipid emulsions on platelet count and coagulation

Platelet Test	Lipid Emulsion	Population	Duration	Main Platelet-Related Outcome	Reference
Platelet Count	SO (100% LCT) (10% or 20%)	Adult patients (n = 6)	3 weeks to 3 months	Mild thrombocytopenia was occasionally reported Large morphologically abnormal platelets	^(50)^
		Children, long-term home PN for short bowel syndrome (n = 7)	3-18 months	Recurrent thrombocytopenia episodes with low platelet counts ↓ platelet survival	^(51)^
		Children (variety of underlying conditions) (n = 29)	> 2 months	Thrombocytopenia Resolved thrombocytopenia with reduced lipid infusion rate (some patients)	^(52)^
		Neonates, infants, children and adolescents (wide range of clinical diagnoses) (n = 180)	1-6 weeks (mean duration 19 days)	= platelet count 10 children with established thrombocytopenia platelet counts rose in all of the cases	^(55)^
		Premature infants (n = 20)	48 h or 4 weeks	= platelet count both in the short (48 h) and long term (4 weeks)	^(54)^
		Newborns (n = 51)	7 days	= platelet count (lipid dose up to 3 g/kg/day)	^(56)^
		Healthy donors (n = 20)	infusion 5 h after overnight fasting	= platelet counts pre- and post-infusion.	^(33)^
		Healthy donors (n = 10)	3 h	= platelet count (during and after the infusion)	^(35)^
	MCT/LCT (50%/50%) or SO (100% LCT) (20%)	Patients (inflammatory bowel disease) (n = 20) Healthy controls (n = 23)	7-10 days	= platelet count between pre- and post-PN (both lipid groups) ↓ platelet count at the end of PN, NS (both lipid groups)	^(30)^
		Cholestasis onset in children receiving long-term PN (n = 183)	3-176 months	↓ platelet count Normalization of platelet count occurred in most cases within the first month after suspension of lipid infusions	^(69)^
	MCT/LCT (50%/50%)	Case report (adult patient) (n = 1)	3 days	Thrombocytopenia, resolved after discontinuation of lipid emulsion	^(70)^
	FO (100%) + SO (100% LCT) or SO (100% LCT) (10%)	Patients (gastrointestinal surgery) (n = 44)	5 days	= platelet count or function (RTG- p-time)	^(83)^
	MCT/OO/FO (30%/25%/15%) (20%)	Case report (paediatric patient) (n = 1)	>30 min	Thrombocytopenia, low fibrinogen levels and coagulopathy (treated by platelet transfusion)	^(93)^
		Postoperative liver transplant patients (n = 54)	30 days	↑ platelet counts 7 days after PN - MCT/OO/FO, = platelet counts days 14 and 30 between lipid groups	^(95)^
	MCT/OO/FO (30%/25%/15%) (20%) or SO (100% LCT) (20%)	Intestinal failure patients (n = 73)	4 weeks	Platelet count was stable throughout the study and no significant changes between lipid groups	^(94)^
Coagulation Tests	SO (100% LCT) (10% or 20%)	Paediatric patients (n = 15)	Average 35.6 months of PN (3-101 months)	↑ PT and PTT Coagulation factor V, fibrinogen remained in normal range	^(58)^
		Case study (7-yr-old patient with hollow viscus myopathy)	Infusion over a 14 h period – 3.3 g fat/kg/day	Haematemesis reported after 6 weeks of PN Giant platelets on smear Prolonged bleeding time PT and PTT associated with platelet dysfunction Fat overload syndrome (lipemic)	^(59)^
	FO (100%) or SO (100% LCT) (10%)	Children with IFALD (n = 262)	PN ≥ 14-30 days	↓ risk of bleeding in FO (100%) patients Bleeding was reported in patients with higher baseline direct of conjugated bilirubin	^(53)^
	FO (100%) + SO (100% LCT) then, FO (100%)	Case study (infant patient with intestinal failure) (n = 1)	26 days of FO (100%) sole therapy	Bleeding after 26 days of FO (100%) treatment No evidence of clot formation, normal PTT Haemorrhage resolved on day 3 with clot formation at bleeding sites	^(84)^
	FO (100%)	Patients going under coronary artery bypass graft surgery (n = 40)	Pre-op IV	= PTT	^(34)^
	MCT/LCT (50%/50%) (20%)	Intestinal failure patients and healthy donors (n = 15)	Average 47.9 days of PN (30-92 days)	= PT, PTT, fibrinogen, TT	^(64)^
	MCT/LCT (50%/50%) or SO (100% LCT) (20%)	Patients (haematologic malignancies, haematopoietic peripheral blood stem cell transplantation) (n = 36)	median of 7 days (range, 4–12)	PT, PTT and fibrinogen levels did not show any difference between lipid groups	^(71)^
	MCT/FO (20%/4%)	Healthy subjects (n = 6)	in vitro study (4, 10 or 25%)	= PT and PTT	^(13)^
	MCT/OO/FO (30%/25%/15%) (20%)	Postoperative liver transplant patients (n = 54)	30 days	= PT and PTT between lipid groups Improved coagulation profile after PN	^(95)^
Platelet Engraftment	SO (100% LCT) (10% or 20%)	Patients with haematologic malignancies (n = 30)	2-27 days	↓ time to platelet engraftment in the individualized PN group than in the conventional PN group	^(63)^
		Haematopoietic stem cell transplantation (n = 61)	app. 12 days	Delay in platelet engraftment ↑ platelet transfusion requirement	^(62)^
	MCT/LCT (50%/50%) or SO (100% LCT) (20%)	Patients (haematologic malignancies, haematopoietic peripheral blood stem cell transplantation) (n = 36)	median of 7 days (range, 4–12)	= platelet engraftment between the lipid groups	^(71)^

SO, soybean oil; LCT, long-chain triglycerides; AA, arachidonic acid; MCT, medium-chain triglycerides; DGLA, dihomo-gamma-linolenic acid; LA, linoleic acid; PN, parenteral nutrition; GPIIb/IIIa, integrin αIIbβ3; FO, fish oil; GPIb, glycoprotein Ib-V-IX; IV, TRAP, thrombin receptor activator peptide; ADP, adenosine diphosphate; OO, olive oil; IV, intravenous; PFA, platelet function analyser; PG, prostaglandin.

↑, increase; ↓, decrease; =, no change; ≤, reduce.

Coagulation tests are frequently used clinical tests to assess blood clotting function in patients^(57)^. The steps in the coagulation cascade are measured by prothrombin time (PT), partial thromboplastin time (PTT), and thrombin time (TT). They can be used to provide information about blood clotting and thrombosis; however, they cannot predict the occurrence of bleeding^(57)^. A study conducted with paediatric patients reported that administration of SO (100% LCT) PN significantly increased PT and PTT after 1 year follow-up. Nevertheless, coagulation factor V and fibrinogen remained in their usual range, which was attributed to vitamin K deficiency^(58)^. A case study reported prolonged bleeding time (PT, PTT) and haematemesis in a patient that received SO (100% LCT) PN, which was due to the developed fat overload syndrome of the high lipid dose; however, the exact aetiology of platelet dysfunction is not known^(59)^.

Platelet engraftment is defined as ‘the independence from platelet infusion for at least seven days, during which the platelet count is regularly measured’ (more than 20 × 10^9^/L)^(60)^ and is known to be crucial for the success of transplantation^(61)^. Platelet engraftment timing is a valid predictor of possible complications in transplantation patients^(61)^, and PN is used to quicken engraftment in these patients^(62,63)^. Studies have shown that individualized PN compared to conventional PN^(63)^ and SO (100% LCT) total PN compared to partial PN in patients with delayed platelet engraftment^(62)^. These results suggest that SO (100% LCT) may protect against thrombocytopenia in transplant patients; however, this may be related to thrombopoiesis.

To conclude, many studies report that SO (100% LCT) lipid emulsion administration is safe^(13,33,35,40,41,54,64)^. Studies demonstrate the potential for increased platelet aggregation^(33,44)^, and this effect may be attributed to the modified fatty acid composition of the platelet plasma membrane^(41,45,46)^. On the other hand, in other studies, it has been reported that SO (100% LCT) may reduce platelet aggregation^(36,37,43)^, which is associated with reduced levels of free cholesterol in platelets^(37)^. The phytosterols in SO (100% LCT) were found to be potentially responsible for thrombocytopenia, leading to bleeding. However, it is essential to note that the observed bleeding was primarily associated with other factors, including PN-related liver disease^(52,53)^, developed fat overload syndrome reported in a case study^(59)^. Moreover, the SO (100% LCT) lipid emulsion should be applied carefully in paediatric patients at risk of bleeding^(53)^. In conclusion, the variability of *in vitro* methods used to test platelet activation^(30)^, as well as differences in the amounts and durations of the lipid emulsions applied^(33,40)^, makes it hard to compare all studies. Accordingly, given that exact aetiology of platelet dysfunction remains unknown, sensitive platelet function tests should be used to investigate SO (100% LCT) lipid emulsions.

### Soybean oil/medium-chain triglycerides-based lipid emulsion

#### Impact on platelet function

Soybean oil/MCT-based lipid emulsions are frequently studied to compare their effects with SO (100% LCT) lipid emulsions (Fig. 2). A small number of studies have demonstrated that platelet aggregation did not change in patients receiving MCT/LCT (50%/50%) emulsions compared to LCT (SO (100% LCT))^(30,42)^. Because the lipid emulsion may lead to changes in the cell membrane fatty acid composition of platelets due to the different fatty acid composition, MCT/LCT (50%/50%) is theoretically proposed to affect platelets differently compared to LCT (SO (100% LCT))^(30,42)^. However, the length of the study might be an essential factor for the modification of platelet membranes, and short study periods might be associated with similar aggregation responses^(42)^. Additionally, the platelet function analyser system (PFA-100) is a new standard to detect platelet dysfunction. It reports a closure time of an easily measured and formed platelet plug under high shear flow conditions^(65)^. A study in healthy male volunteers showed no difference in platelet function via the PFA-100 system due to the lipid emulsion MCT/LCT (50%/50%), indicating it is safe to use^(65)^ (Table 4).

**Table 4.** Summary of the studies measuring the effect of soybean oil/medium-chain triglycerides-based lipid emulsions on platelet function

Platelet Test	Population	Duration	Main Platelet-Related Outcome	Reference
Platelet Structure	Critically ill adult patients (n = 12)	7 days	≤ percentage of palmitoleic acid and AA	^(45)^
	Patients with malnutrition (n = 20)	8-10 days	↓ oleic acid percentage and AA ↑ palmitic acid, total SFAs, DGLA Lauric and myristic acids not detected in the platelet phospholipids	^(46)^
	Patients in need of long-term home PN (n = 42)	long-term home PN (min 8 weeks)	= proportion of n-3 and n-6 fatty acids	^(72)^
	Normolipidemic subjects (n = 8)	5 h infusion, >4 days	↑ LA concentration	^(68)^
Platelet Activation	Intestinal failure patients (n = 15), healthy donors (control) (n = 15)	Average 47.9 days of PN (30-92 days)	↑ P-selectin expression – patients = GPIIb/IIIa expression	^(64)^
	Male volunteers (n = 12)	bolus IV injection (50 mL)	No adverse effects (platelet reactivity, closure time by ADP or epinephrine) by GPIIb/IIIa, P-selectin, fibrinogen, GPIb expression	^(67)^
	Male volunteers (n = 12)	bolus IV injection (50 mL)	= GPIb, P-selectin, fibrinogen, GPIIb/IIIa expression	^(65)^
	Healthy donors (n = 20)	*ex vivo* platelet activation	↑ expression of GPIIb/IIIa and P-selectin ↓ (slightly) expression of GPIb (TRAP-6 and ADP and no activation)	^(32)^
Platelet Aggregation	Patients (inflammatory bowel disease) (n = 20) Healthy controls (n = 23)	8-10 days	= platelet aggregation of patient’s pre- and post-PN = platelet aggregation post-SO (100% LCT) and post-MCT/LCT (50%/50%) = platelet aggregation pre-PN and post-PN.	^(30)^
	Critically ill adult patients (n = 23)	7 days	= platelet aggregation of the groups of PN	^(42)^
PFA-100 System	Male volunteers (n = 12)	bolus IV injection (50 mL)	PFA-100 system: = closure time of ADP and epinephrine tests	^(65)^
Platelet Activation Parameters	Critically ill adult patients (n = 23)	7 days	↑ PF4 (platelet-activating factor 4) and βTG (beta-thromboglobulin) continued to be elevated at 4-7 days of PN	^(42)^
Lipid Mediator Profile	Critically ill adult patients (n = 23)	7 days	↓ 6-keto-PGF_1α_ values after 4-7 days, NS	^(42)^

SO, soybean oil; LCT, long-chain triglycerides; AA, arachidonic acid; MCT, medium-chain triglycerides; DGLA, dihomo-gamma-linolenic acid; LA, linoleic acid; PN, parenteral nutrition; GPIIb/IIIa, integrin αIIbβ3; FO, fish oil; GPIb, glycoprotein Ib-V-IX; IV, TRAP, thrombin receptor activator peptide; ADP, adenosine diphosphate; OO, olive oil; IV, intravenous; PFA, platelet function analyser; PG, prostaglandin.

↑, increase; ↓, decrease; =, no change.

Flow cytometry is used as a sensitive tool for platelet function^(66)^; however, studies regarding the effects of parenteral lipid emulsions on platelet aggregation and expression of GPIb, GPIIb/IIIa, and P-selectin are limited. The upregulation of platelet membrane glycoproteins may be associated with coagulopathy induced by PN^(64)^. *In vivo* studies have shown that MCT/LCT (50%/50%) administration did not show any difference in the expression of platelet receptors GPIIb/IIIa^(64,65,67)^, P-selectin^(65,67)^, fibrinogen^(65,67)^, and GPIb^(65,67)^ against agonists ADP, collagen, and TRAP-6^(67)^ in patients^(64)^ and healthy donors^(65,67)^. However, platelet P-selectin expression of the PN group was significantly higher than that of the control group (healthy donors) following long-term PN with MCT/LCT (50%/50%)^(64)^. Thus, PN administration longer than 30 days induced the activation of platelet glycoproteins, which may be a risk factor for thrombogenesis^(64)^. The fact that there was no platelet activation in bolus IV lipid emulsion injections to healthy donors^(65,67)^ raises the idea that the metabolic state of patients and their potential response to PN lipid emulsion treatment may be an additional factor to keep in mind. Moreover, Stoetzer et al. demonstrated that MCT/LCT (50%/50%) lipid emulsion increased the GPIIb/IIIa and P-selectin expression while decreasing the GPIb expression in *ex vivo* studies involving healthy donors^(32)^. This study emphasizes that their findings cannot be interpreted into *in vivo* conditions; nonetheless, it highlights the potential for PN to interact with cellular coagulation processes^(32)^ (Table 4).

Similarly, the platelet membrane fatty acids were investigated in patients receiving lipid emulsions, including MCT/LCT (50%/50%). The MCT/LCT (50%/50%) lipid emulsion reduced AA^(45,46)^ and palmitoleic acid^(45)^, whereas it increased the percentage of linoleic acid^(68)^, palmitic acid, SFAs^(46)^, and dihomo-γ-linolenic acid (DGLA)^(46)^ in platelets. Interestingly, platelet oleic acid content decreased, and lauric acid and myristic acid were not detected in platelet phospholipids^(46)^. Additionally, plasma composition alterations were reported in response to MCT/LCT (50%/50%) lipid emulsion administration. In particular, there was an increase in the percentage of LA, palmitoleic, and palmitic acid, while oleic acid decreased (Table 4). Moreover, low levels of plasma lauric and myristic acids were reported following the MCT/LCT (50%/50%) administration^(46)^, which is probably in response to the lipid source coconut oil^(4)^ (Table 1).

Platelet-related mediators were analysed in patients treated with lipid emulsions MCT/LCT (50%/50%). A study reported that the release of prostacyclin (measured by stable metabolite 6-keto-PGF_1α_) decreased in patients receiving MCT/LCT (50%/50%), although the difference was not statistically significant^(42)^. Additionally, the administration of MCT/LCT (50%/50%) to patients resulted in a continuous elevation of PF4 and βTG in activated platelets at 4-7 days of PN treatment; however, no significant difference was observed when compared to the lipid emulsion SO (100% LCT)^(42)^. These activation markers may be affected by the oxygen radicals in critical patients and thus showed no alterations in platelet function^(42)^ (Table 4).

#### Impact on platelet count and coagulation

A study involving patients receiving parenteral nutrition lipid emulsions MCT/LCT (50%/50%) or SO (100% LCT) revealed no significant difference in platelet counts between pre- and post-PN administration^(30)^. Conversely, a study on children receiving long-term PN with either MCT/LCT (50%/50%) or SO (100% LCT) lipid emulsions reported a reduction in platelet count. Interestingly, normalization of platelet count occurred in some cases within the first month after suspension of lipid infusions. As a result, this study could not report a protective effect of MCT/LCT (50%/50%) emulsions against hepatic injury and cholestasis, which may develop with the long-term infusion of lipid emulsions even within lipid infusion rate limits^(69)^. While lipid emulsion-induced thrombocytopenia may be an adverse effect in paediatric patients, a case study involving an adult patient showed thrombocytopenia following lipid emulsion MCT/LCT (50%/50%) administration, which may be because of the pre-existing malnutrition of the patient^(70)^. On the other hand, both healthy donors and patients who received the lipid emulsion MCT/LCT (50%/50%) showed no change in the coagulation tests, including PT, PTT, fibrinogen, TT^(64)^. Furthermore, the comparison of MCT/LCT (50%/50%) and SO (100% LCT) lipid emulsions did not differ in coagulation parameters, including PT, PTT, and fibrinogen levels^(71)^ (Table 3).

Similarly, the lipid emulsion MCT/LCT (50%/50%) did not differ in platelet engraftment in patients with various haematologic malignancies who underwent a haematopoietic peripheral blood stem cell transplantation^(71)^. Thus, MCT/LCT (50%/50%) may protect against thrombocytopenia. However, it is also proposed that engraftment duration is shorter in well-nourished patients; therefore, PN instead of the lipid emulsion type may be therapeutic in the context of platelet engraftment^(63)^ (Table 3).

In conclusion, MCT/LCT (50%/50%) lipid emulsion showed no risk of bleeding in patients^(30,64,71)^. Low platelet count was reported in children receiving long-term PN^(69)^. Additionally, increased platelet GPIIb/IIIa^(32)^ and P-selectin^(32,64)^ expression and decreased GPIb expression^(32)^ were shown. However, platelet aggregation^(30,42)^ was not affected by the lipid emulsion MCT/LCT (50%/50%). The increased platelet activation may be due to *ex vivo* results^(32)^ or patient status^(64)^, as platelet reactivity did not change in other studies^(65,67)^. The change in platelet membrane fatty acids as in reduced AA^(45,46)^; however, increased SFAs percentage^(46)^ and increased LA^(68)^ may be a mechanism leading to platelet activation, however, the following production of platelet lipid mediators did not show any change in studies. Moreover, the stable proportion of n-3 and n-6 fatty acids in platelets^(72)^ may represent the underlying mechanism for the protective effects of MCT/LCT (50%/50%) during platelet activation. To conclude, although MCT/LCT (50%/50%) lipid emulsion was reported safe to use towards the risk of thrombosis and bleeding, its protective effects compared to SO (100% LCT) lipid emulsion are not clearly shown in studies.

### Fish oil-based lipid emulsion

#### Impact on platelet function

Pure fish oil-based lipid emulsions were also investigated in the context of platelets (Fig. 2). It has been reported that PN consisting of FO (100%) lipid emulsion decreased the aggregation response of platelets in surgical patients^(34)^. This result was particularly pronounced with collagen aggregation, and there was no risk of bleeding in these patients. Therefore, FO (100%) lipid emulsions may be used to inhibit platelet aggregation, providing a cardioprotective effect in patients undergoing open heart surgery. However, the risk of bleeding should be considered carefully. Moreover, a remarkable reduction in aggregation was reported in healthy subjects following administration of FO (100%) lipid emulsion^(73)^. Following the 24-hour administration of FO (100%) infusion to healthy subjects, collagen-induced aggregation of platelets increased even higher than the starting value, whereas ADP-induced aggregation (20% reduction) remained the same^(73)^. Thus, FO (100%) infusion may have potential use in patients with high thrombosis risk^(73)^. In contrast, IV or oral administration of FO (100%) lipid emulsion to healthy subjects showed no change in platelet function as in platelet adhesion and aggregation tested via a PFA-100 system, which may be due to the short-term study period (15 days)^(74)^ (Table 5).

**Table 5.** Summary of the studies measuring the effect of fish oil-based lipid emulsions on platelet function

Platelet Test	Population	Duration	Main Platelet-Related Outcome	Reference
Platelet Structure	Case study (patient with ulcerative colitis) (n = 1)	9-29 days	↑ EPA, DHA (to a lesser extent) ↓ AA ↑ n-3 fatty acid/AA ratio = 16:0, 18:0, 18:1 and 18:2	^(75)^
	Haemodialysis patients (n = 87)	4 h of haemodialysis	↑ (slightly) DHA (after 4 hours) ↑ EPA, DHA (after 48 hours) ↑ EPA, DHA	^(76)^
	Cardiac surgery patients (n = 28)	3 times as 2 h infusions	↑ platelet EPA and DHA ↑ atrial tissue EPA concentrations	^(77)^
	Healthy male subjects (n = 16)	0.5 g/kg FO twice for 48 h or 24 h	Basal platelet EPA and DHA phospholipids were low ↑ EPA (1.68%) and DHA (3.32%) in platelet phospholipids	^(78)^
	Cystic fibrosis patients (n = 13)	2mL/kg/wk. FO IV emulsion, 12 wks.	↑ platelet EPA and DHA = AA. ↓ n-9 = n-3, n-6, n-9 FA (plasma)	^(79)^
	Oesophageal carcinoma patients (n = 19)	7 days	↑ EPA in both platelet PC and PE ↑ EPA/AA ratios (doubled) in both PC and PE	^(81)^
	Healthy subjects (n = 8)	IV (1 week) or oral SUP (3 days)	↑ platelet membrane EPA, DHA Rapid incorporation of EPA than DHA	^(74)^
Platelet Aggregation	Patients going under coronary artery bypass graft surgery (n = 40)	Pre-op IV	= post-op platelet aggregation by ADP, AA, TRAP ↓ post-op platelet aggregation by collagen = surgical blood loss	^(34)^
	Healthy male subjects (n = 13)	IV injection >60 min	↓ platelet aggregation induced by collagen and ADP in 60 min, till 6 hours.	^(73)^
PFA-100 System	Healthy subjects (n = 8)	IV (1 week) or oral SUP (3 days)	Platelet function by PFA-100 system: = baseline and 15 days and IV and oral SUP	^(74)^
Lipid Mediator Profile	Septic shock patients (n = 10) and healthy controls (n = 8)	10 days	↓ PAF secretion ↑ PAF synthesis over time ↑ TXA_3_ and TXA_3_/TXA_2_ ratio (17.8%)	^(49)^
	Patients with chronic plaque-type psoriasis skin disease (n = 83)	14 days	↑ TXB_3_ generation (a plateau between days 7 and 15) ↓ in TXB_2_ (slight) ↑ EPA (plasma) (a plateau after the 7^th^ day of infusion and dropped to baseline values on day 40) = AA (plasma)	^(80)^
	Case study (patient with ulcerative colitis) (n = 1)	9-29 days	↑ platelet TXB_3_ generation progressively TXB_3_/TXB_2_ ratio approached 0.2 at the end of the period	^(75)^
	Healthy male subjects (n = 13)	IV injection >60 min	↓ TXB content during the infusion (PRP - platelet stimulation with collagen) PGE content varied slightly throughout the whole period	^(73)^

FO, fish oil; EPA, eicosapentaenoic acid; DHA, docosahexaenoic acid; AA, arachidonic acid; IV, intravenous; FA, fatty acids; SUP, supplementation; PC, phosphatidylcholine; PE, phosphatidylethanolamine; SO, soybean oil; ADP, adenosine diphosphate; Pre-op, preoperative; Post-op, postoperative; TRAP, thrombin receptor activator peptide PFA, platelet function analyser; PAF, platelet-activating factor; TX, thromboxane; PRP, platelet rich plasma; PG, prostaglandin.

↑, increase; ↓, decrease; =, no change.

The anti-thrombotic effects of FO (100%) lipid emulsion are suggested to be related to the alterations in plasma membrane phospholipid fatty acid profile and consequent eicosanoid synthesis^(34,73,74)^. In particular, FO (100%) lipid emulsion showed increased EPA and DHA in platelet phospholipids/membrane^(74–80)^ along with decreased AA and increased n-3 PUFA/AA membrane ratios^(75)^. Moreover, a study demonstrated that patients receiving FO (100%) showed a substantial increase in EPA levels in both platelet phosphatidylcholine and phosphatidylethanolamine compared to SO (100% LCT)^(81)^. Additionally, plasma EPA and DHA levels increased several folds^(75,76)^, surpassing AA^(75)^. Conversely, no change was reported in fatty acids palmitic, stearic, oleic, and linoleic acid^(75)^. Notably, a rapid increase in n-3 PUFA in platelet phospholipids was reported within 4 hours during haemodialysis (single dose of n-3 PUFA lipid emulsion)^(76)^ and within 12 hours perioperative administration of FO (100%)^(77)^. Meanwhile, with n-3 PUFA administration, the increase of n-3 PUFA in plasma phospholipids occurred within 48 hours^(76)^. It is important to note that the rapid increase in platelet EPA and DHA was attributed to the exchange between plasma NEFA and platelet fatty acids^(76)^ (Table 5).

The potential of lipid mediators has yet to be fully understood; moreover, their role in platelets in health and disease states encourage new lipidomic methodologies to scan and image lipid mediators^(82)^. In a case study, the administration of IV FO (100%) resulted in an increase in the TXB_3_/TXB_2_ ratio in stimulated platelets during infusion^(75)^. Various studies involving patients or healthy subjects have demonstrated decreased blood TX and TXB_2_ levels and increased blood and platelet-generated TXA_3_ levels^(49,73)^. Moreover, an increase in platelet TXB_3_ generation^(80)^ and elevated TXA_3_/TXA_2_ ratio have been observed^(49)^, while prostaglandin levels remained stable^(73)^. Also, FO (100%) administration increased platelet-activating factors synthesis over time^(49)^. Together, these findings may indicate the influence of the alternative lipid precursor on central lipoxygenase and cyclooxygenase pathways. These findings suggest that some EPA-containing membrane lipid pool(s), providing precursor fatty acids to the metabolic pathways of eicosanoid formation, may be rapidly regulated in exchange with plasma EPA^(80)^ (Table 5).

#### Impact on platelet count and coagulation

Fish oil-based lipid emulsions have been investigated concerning the risk of bleeding (Fig. 2a) and have been reported safe to use, with no evidence suggesting an increased risk of coagulopathy or bleeding abnormalities^(8)^. However, it is essential to recall that the studies are limited. Patients receiving PN formulations with FO (100%) + SO (100% LCT) or SO (100% LCT) were investigated, and no differences were observed in terms of platelet counts or platelet function (measured by RTG- p-time) between groups^(83)^. Alternatively, bleeding was reported in a patient with intestinal failure after 26 days of sole FO (100%) lipid emulsion administration, following a regimen of FO (100%) + SO (100% LCT) lipid emulsion. Besides, there was no evidence of clot formation, PTT was normal, and haemorrhage resolved on day 3 with clot formation at bleeding sites^(84)^. It has been reported that preoperative IV FO (100%) did not induce a change in PTT among surgical patients^(34)^. Given this data, it was suggested that the potential for bleeding complications from sole FO-based therapy in high-risk infants with liver disease should be reconsidered^(84)^ (Table 3).

In contrast, a study showed that FO (100%) lipid emulsion had a lower risk of bleeding in children with IFALD compared to SO (100% LCT) lipid emulsion, which was associated with bleeding. In theory, FO-based lipid emulsions have an increased risk of bleeding due to their inhibitory effects in platelet adhesion and platelet-stimulated thrombin generation. However, this might be a weak effect due to this study. Nevertheless, due to the lack of data, there should be caution when using FO-based lipid emulsions, especially in paediatric patients at risk of bleeding^(53)^.

In conclusion, the demonstrated anti-inflammatory effects of fish oil endorse the FO-based lipid emulsions as advantageous components in PN^(2,3,6,7,12,85)^. The proposed reduction in platelet aggregation^(73)^ after FO (100%) administration may result from two possible mechanisms. Firstly, the inhibition of cyclooxygenase by n-3 PUFAs, leading to decreased synthesis of TXA_2_ from AA in platelets^(34,84)^. Secondly, it could be a result from the synthesis of TXA_3_ from EPA or a general reduction in total TX synthesis^(49,73)^. In the end, leading to elevated levels of TXB_3_^(80)^ and TXA_3_^(73)^, thus elevated ratios of TXB_3_/TXB_2_^(75)^ and TXA_3_/TXA_2_^(49)^. In addition, this may be supported by the changes in platelet membrane structure, characterized by an increase in EPA and DHA^(75,76,79,81)^ along with decreased AA in membrane phospholipids. Moreover, an increase in n-3 PUFA/AA membrane ratios in the platelet membrane was reported^(75)^. Alternatively, a very low AA source may potentially result in AA inhibition that has been found to block platelet shape change and granule secretion, thus decreasing platelet aggregation^(84)^. However, there might be a temporary inhibition of platelet aggregation due to FO (100%) lipid emulsion; hence, it may be favourable in high thrombosis risk patients^(73)^. However, the acute effects of n-3 PUFAs might not be mediated by platelet plasma membrane incorporation since there was no change in platelet function via the PFA-100 system^(74)^, and other alternative lipid precursor pools are pointed out^(80)^. Additionally, the reported anti-platelet effects were not shown to cause bleeding risk in many studies^(8,53)^, except a case report^(84)^ indicating that this lipid emulsion should be carefully used in paediatric patients^(53)^. Although n-3 fatty acids may likely affect haemostasis^(86)^, The American Society for Parenteral and Enteral Nutrition states that there is no evidence to support that fish oil-containing lipid emulsions increase the risk of coagulopathy or bleeding abnormalities^(8)^.

### Olive oil/soybean oil-based lipid emulsion

#### Impact on platelet function

The evidence in the literature on the effects of OO/SO (80%/20%) lipid emulsion is limited, with only one *ex vivo* study related to the topic found (Fig. 2). Flow cytometry, a sensitive tool for platelet function^(66)^, was used to examine the effects of OO/SO (80%/20%) lipid emulsion on platelet function. Previous research indicated that the stimulation of platelets of healthy donors by OO/SO (80%/20%) incubation did not influence GPIIb/IIIa, P-selectin, and GPIb receptor expression independent of lipid concentration and stimulation of ADP and TRAP-6^(32)^. A study suggests that olive oil-based lipid emulsion OO/SO (80%/20%) has superior anti-inflammatory effects when compared to the blend lipid emulsion MCT/OO/FO (30%/25%/15%)^(87)^. Therefore, its effects on platelets should be further investigated, including whether it may be used in patients with high thrombosis or bleeding risk (Table 6).

**Table 6.** Summary of the studies measuring the effect of olive oil/soybean oil-based lipid emulsions on platelet function

Platelet Test	Lipid Emulsion	Population	Duration	Main Platelet-Related Outcome	Reference
Platelet Activation	MCT/LCT (50%/50%) or MCT/FO (50%/10%) or OO/SO (80%/20%) (20%)	Healthy donors (n = 20)	*ex vivo* platelet activation	= expression of GPIIb/IIIa, P-selectin, GPIb (TRAP-6 and ADP and no activation)	^(32)^

MCT, medium-chain triglycerides; LCT, long-chain triglycerides; OO, olive oil; SO, soybean oil; FO, fish oil.

=, no change.

### Medium-chain triglycerides/fish oil-based lipid emulsion

#### Impact on platelet function

Studies investigating the lipid emulsion MCT/FO (50%/10%) are also limited in the literature (Fig. 2). In PN solutions, including MCT/FO (20%/4%) that were added to healthy subjects’ plasma in different concentrations of volume (4, 10, 25%), platelet aggregation was decreased at a concentration of 4%, and the pore closure time was prolonged, indicating platelet function inhibition^(13)^. Compared to the lipid emulsion MCT/LCT (50%/50%) in healthy male volunteers, both emulsions showed no difference in platelet function via the PFA-100 system. Although the overall closure time was lower than basal values by MCT/FO (80%/20%) and MCT/LCT (50%/50%), it is indicated that both lipid emulsions are safe to use in the context of platelet adhesion and aggregation^(65)^ (Table 5).

The platelet function after MCT/FO (80%/20%) lipid emulsion administration was further assessed using flow cytometry. Since MCT is a substrate for lipoprotein lipase hydrolysis, the MCT/FO (80%/20%) lipid emulsion is proposed to serve as a rapid source of released MCT and n-3 fatty acids from emulsion particles, which subsequently incorporate into platelet cell membranes^(65,67)^. The n-3 fatty acid availability may be associated with decreased platelet aggregation and increased bleeding risk, which raises safety concerns regarding using MCT/FO (80%/20%) lipid emulsion^(65)^. A previous report indicated that MCT/FO (80%/20%) administration to healthy subjects resulted in a lower P-selectin expression on day two by collagen^(65)^. Nevertheless, after a bolus IV injection of MCT/FO (80%/20%) to healthy subjects, no significant changes in the expression of platelet receptors GPIIb/IIIa^(65,67)^, P-selectin^(65,67)^, fibrinogen^(65,67)^, and GPIb^(65,67)^ were observed in response to agonists ADP, collagen, and TRAP-6^(67)^. These bolus IV studies have concluded that MCT/FO (80%/20%) lipid emulsions are safe to use and may show cardioprotective effects. An *ex vivo* study using the MCT/FO (50%/10%) (0.6 mg/ml) lipid emulsion on healthy subjects increased the expression of GPIIb/IIIa and P-selectin and, by contrast, decreased the expression of GPIb, indicating platelet activation^(32)^. However, these results indicate that *in vivo* outcomes may differ (Table 7).

**Table 7.** Summary of the studies measuring the effect of medium-chain triglycerides/fish oil-based lipid emulsions on platelet function

Platelet Test	Population	Duration	Main Platelet-Related Outcome	Reference
Platelet Structure	Patients in need of long-term home PN (n = 42)	long-term home PN (min 8 weeks)	↑ EPA, DHA, DPA (platelet, serum) ↓ LA, DGLA, GLA (platelet) ↓ LA, AA (serum)	^(72)^
	Normolipidemic subjects (n = 8)	5 h infusion, >4 days	= LA, DHA ↓ AA concentration (slightly) ↑ EPA (day 2)	^(68)^
	Male volunteers (n = 12)	5 min bolus IV injection (50 mL)	↑ plasma non-esterified fatty acid concentration (fast plasma elimination of lipid emulsion) ↑ content of n-3 PUFAs, EPA (platelet phospholipids) within 60 min and >24 h	^(88)^
Platelet Activation	Male volunteers (n = 12)	bolus IV injection (50 mL)	No adverse effects (platelet reactivity, closure time by ADP or epinephrine) by GPIIb/IIIa, P-selectin, fibrinogen, GPIb expression	^(67)^
	Male volunteers (n = 12)	bolus IV injection (50 mL)	= GPIb, P-selectin, fibrinogen, GPIIb/IIIa expression ↓ p-selectin expression on day 2 MCT/FO by collagen	^(65)^
	Healthy donors (n = 20)	*ex vivo* platelet activation	↑ expression of GPIIb/IIIa and P-selectin – MCT/FO (50%/10%) ↓ expression of GPIb (slightly) (TRAP-6 and ADP and no activation)	^(32)^
Platelet Aggregation	Healthy subjects (n = 6)	in vitro study (4, 10 or 25%)	↓ platelet aggregation already in 4% MCT/FO (20%/4%) Prolonged pore closure time indicating platelet function inhibition	^(13)^
PFA-100 System	Male volunteers (n = 12)	bolus IV injection (50 mL)	PFA-100 system: No difference between lipid emulsions ↓ overall closure time by MCT:FO in response to ADP (lesser than MCT/LCT)	^(65)^
Lipid Mediator Profile	Patients with metastatic pancreatic cancer (n = 32)	3 weeks/1 weeks rest	≤ cytokine platelet-derived growth factor (PDGF) and fibroblast growth factor (FGF)	^(89)^

MCT, medium-chain triglycerides; FO, fish oil; LA, linoleic acid; DHA, docosahexaenoic acid; AA, arachidonic acid; LCT, long-chain triglycerides; EPA, eicosapentaenoic acid; PN, parenteral nutrition; DPA, docosapentaenoic acid; DGLA, dihomo-gamma-linolenic acid; GLA, gamma-linolenic acid; IV, intravenous; ALA, α-Linolenic acid; GPIIb/IIIa, integrin αIIbβ3; GPIb, glycoprotein Ib-V-IX; TRAP, thrombin receptor activator peptide; ADP, adenosine diphosphate; OO, olive oil; SO, soybean oil; PFA, platelet function analyser.

↑, increase; ↓, decrease; =, no change; ≤, reduce.

The lipid emulsion MCT/FO (50%/10%) compared to MCT/LCT (50%/50%) emulsion increased EPA^(67,68,72,88)^, DHA^(72)^, and DPA^(72)^, whereas decreased AA^(67,68)^, LA^(67,72)^, DGLA^(72)^, γ-linolenic acid (GLA)^(72)^ in platelets. Also, other studies reported no significant effects on the levels of LA, DHA^(68)^, ALA, and docosapentaenoic acid (DPA)^(67)^ in platelet phospholipids. In addition, the MCT/FO (50%/10%) emulsion was found to have a greater plasma elimination in the NEFA concentration content compared to MCT/LCT (50%/50%). Accordingly, platelets are rapidly enriched with n-3 PUFAs after bolus IV MCT/FO (50%/10%) injection^(88)^. Additionally, MCT/FO (50%/10%) lipid emulsion-treated patients showed reduced platelet-derived growth factor, along with medical treatment^(89)^ (Table 7).

### Soybean oil/medium-chain triglycerides/olive oil/fish oil-based lipid emulsion

#### Impact on platelet function

The lipid emulsion MCT/OO/FO (30%/25%/15%) is discussed in the literature due to its varying lipid sources and different fatty acids (Fig. 2). However, there is a limited number of studies related to its effects on platelets. When compared to SO (100% LCT), the MCT/OO/FO (30%/25%/15%) lipid emulsion increased total n-3 PUFA, EPA, DHA levels, n-3/n-6 PUFA and EPA/AA ratios, decreased total n-6 PUFA, and did not change AA in plasma phospholipids in patients receiving PN for over five days. This change in plasma fatty acids was also reported as similar to changes in platelet phospholipid composition^(90)^. These findings may be attributed to its n-6:n-3 PUFA ratio, 2.5:1^(10)^ (Table 1). The plasma lipid mediator profile after PN, including MCT/OO/FO (30%/25%/15%), showed increased leukotriene B_5_ and not significantly decreased leukotriene B_4_ with increased plasma leukotriene B_5_/B_4_ ratio in the plasma, suggesting favourable anti-inflammatory effects. However, platelet responses to this environment were not investigated^(90)^. Thus, the proposed anti-inflammatory environment due to MCT/OO/FO (30%/25%/15%) lipid emulsion should be investigated in platelets and thrombus formation.

#### Impact of blend lipid emulsions on platelet count and coagulation

The blend lipid emulsion MCT/OO/FO (30%/25%/15%) has been investigated in the literature and is shown that it may have benefits over the first-generation lipid emulsion SO (100% LCT)^(91,92)^; however, platelet-related studies are limited (Fig. 2a). According to a case report, thrombocytopenia was observed in a paediatric patient, with short bowel syndrome and IFALD, receiving a very rapid infusion of PN including MCT/OO/FO (30%/25%/15%) lipid emulsion^(93)^ (Table 3). It was reported that the lipid composition is also essential as fish oil-based lipid emulsions may not lead to the fat overload syndrome due to an n-6:n-3 PUFA ratio of approximately 2.5:1 in contrast to soybean oil lipid emulsions, which has a ratio of 7:1 (Table 1). Thus, this case study confirms the infusion rate’s importance before the solution’s lipid composition^(93)^. On the other hand, the lipid emulsion MCT/OO/FO (30%/25%/15%) did not increase the risk of thrombocytopenia in long-term patients^(94,95)^ and did not change PT and PTT^(95)^; thus, it was reported safe without increased coagulation risk and bleeding^(94,95)^ (Table 3). Moreover, the lipid emulsion MCT/FO (20%/4%) administered to healthy subjects at different concentrations of plasma did not change PT and PTT^(13)^ (Table 3), and OO/SO (80%/20%) lipid emulsion was not investigated in the literature on platelet count and coagulation (Fig. 2).

To conclude, the PN lipid emulsion MCT/FO (20-50%/4-10%) might decrease platelet aggregation, indicating platelet function inhibition; however, there was no change in PT and PTT^(13)^. In addition, platelet activation was reported by the increased receptor expression of GPIIb/IIIa and P-selectin and decreased GPIb^(32)^ *ex vivo*; however, no change of the expressions of GPIIb/IIIa, P-selectin, fibrinogen, and GPIb were shown *in vivo* ^(67)^. The observed increases in the levels of EPA^(67,68,72,88)^, DHA^(72,88)^, DPA^(72)^, as well as decreases in the levels of AA^(67,68)^, LA^(67,72)^, DGLA, GLA^(72)^ and platelet-derived growth factor^(89)^ may explain the reduction in platelet aggregation in response to MCT/FO (20-50%/4-10%). Overall, different concentrations of MCT/FO administration in PN solutions were reported safe regarding platelet activation processes^(65)^. However, there are only limited studies to conclude that it may inhibit/increase platelet activation and platelet aggregation (Fig. 2a).

Furthermore, the literature lacks data about blend parenteral nutrition lipid emulsions OO/SO (80%/20%) and MCT/OO/FO (30%/25%/15%) on platelets. The lipid emulsion OO/SO (80%/20%) demonstrated no change in platelet activation assessed by flow cytometry and was reported as a safe formula to use^(32)^. Moreover, MCT/OO/FO (30%/25%/15%) lipid emulsion was also reported safe to use without increased coagulation risk and bleeding^(94,95)^; however, platelet activation and aggregation along with changes in platelet lipids and the potentially synthesized lipid mediators were not investigated. According to the limited literature, both fish oil blend lipid emulsions may be advantageous in patients who have a high risk of bleeding^(13,95)^, yet this subject should be further investigated. The proposed anti-inflammatory effects of these blend lipid emulsions should be further investigated in platelets to define their protocol in patients with high thrombosis or bleeding risk (Table 6).

### Conclusions and future directions

According to guidelines, lipid emulsions that are essential macronutrient components of parenteral nutrition solutions, are generally found safe to use in bleeding and thrombotic disease processes. Potentially, lipid emulsions may be responsible for platelet-related changes by altering the plasma and cell membrane fatty acid composition and further eicosanoid synthesis. This may lead to changes in the platelet plasma membrane cholesterol/phospholipid ratio, which is an important determinant of membrane fluidity. Altered membrane fluidity might change the permeability and behaviour of membrane-bound enzymes and the receptor activity of platelets. The cell membrane structure is reported to be modified by PN with lipid emulsions.

Further, the plasma levels of NEFA may, in turn, activate platelets directly, leading to bleeding or thrombotic tendency. The lipid emulsions may also contain fatty acids (arachidonic acid), which might activate platelets directly. Additionally, the development of fat accumulation as the fat overload syndrome with high serum lipid levels may result in haematologic symptoms such as prolonged bleeding time and decreased platelet survival.

The reviewed papers on parenteral nutrition lipid emulsions and their effects on platelets pointed out patients at risk of bleeding and platelet aggregation. A limited number of studies have mentioned that some lipid emulsions (SO (100% LCT), FO (100%)) were reported to lead to bleeding in patients, which may be associated with other parenteral nutrition or patient-related factors. However, these lipid emulsions should be used cautiously, especially in paediatric patients with parenteral nutrition-related liver disease. In this context, due to the risk of decreased platelet aggregation response by FO (100%) lipid emulsion, FO blend lipid emulsions may be advantageous. However, there is only a limited number of studies investigating their effects on platelets. The inconsistency of studies is principally due to the methodological differences of the studies (platelet function tests, *in vivo/ex vivo* study design), patient characteristics (adult/paediatric, age, comorbidities), etc.

Furthermore, the results from patients and healthy subjects exclude the status of critically ill patients and their affected haemostasis due to other pathological factors. However, the investigated studies enhance the assumption that lipid emulsions affect platelets differently. Studies investigating platelets and parenteral nutrition should be supported to minimize the adverse effects and to benefit from the potential protective effects of parenteral nutrition lipid emulsions on platelets.

To conclude, it is now well known that the membranes of platelets that provide substrates for enzymatic conversion, can change in health and disease states and are responsive to the diet. Additionally, dietary intake of lipids and fatty acids is an evolving area of research in many diseases and health. Thus, the *in vivo* nature of parenteral nutrition therapy benefits future studies to investigate the effects of dietary lipids and fatty acids on diseases, health, and human metabolism.

## Abbreviations

**AA**: arachidonic acid; **ADP**: adenosine diphosphate; **ALA**: α-linolenic acid; **DGLA**: dihomo-γ-linolenic acid; **DHA**: docosahexaenoic acid; **DPA**: docosapentaenoic acid; **EPA**: eicosapentaenoic acid; **FO**: fish oil; **GP**: glycoprotein; **GPIb**: glycoprotein Ib-V-IX; **GPIIb/IIIa**: integrin αIIbβ3; **IFALD**: intestinal failure-associated liver disease; **IV**: intravenous; **LA**: linoleic acid; **LCT**: long-chain triglycerides; **MCT**: medium-chain triglycerides; **MUFA**: monounsaturated fatty acids; **NEFA**: non-esterified fatty acids; **OO**: olive oil; **PFA**: platelet function analyser; **PN**: parenteral nutrition; **PS**: phosphatidylserine; **PT**: prothrombin time; **PTT**: partial thromboplastin time; **PUFA**: polyunsaturated fatty acids; **SO**: soybean oil; **TEG**: tromboelastography; **TG**: triglyceride; **TRAP**: thrombin receptor activator peptide; **TT**: thrombin time; **TX**: thromboxane.
